# The Spectrum of Hypogonadism in Women From Basrah

**DOI:** 10.7759/cureus.67990

**Published:** 2024-08-28

**Authors:** Alyaa K Zuhairi, Ammar M Almomin, Emad Alhubaish, Abbas A Mansour

**Affiliations:** 1 Endocrinology and Diabetes, Faiha Specialized Diabetes, Endocrine and Metabolism Center, University of Basrah, Basrah, IRQ

**Keywords:** disorders of sex development, hypogonadotropic hypogonadism, hypergonadotropic hypogonadism, amenorrhea, female hypogonadism

## Abstract

Background

Determining the causes of female hypogonadism is crucial for guiding management and preventing complications. This study aimed to categorize the causes of female hypogonadism in Basrah and identify its frequency.

Methodology

This retrospective single-center study analyzed 1,111 women diagnosed with hypogonadism between 2008 and 2024 and described its etiology in women less than 45 years old (before menopause). The study was conducted in the Faiha Specialized Diabetes, Endocrine and Metabolism Center in Basrah, southern Iraq. Cases were classified into hypogonadotropic hypogonadism and hypergonadotropic hypogonadism according to specific causes such as disorders of sex development or difference (DSDs).

Results

The most frequent etiology in the 1,111 patients was hypogonadotropic hypogonadism, documented in 844 (76%) cases; functional amenorrhea was predominant in 402 (47.63%) of them. Next were 218 (20%) cases of hypergonadotropic hypogonadism. DSDs were documented in a small percentage of female hypogonadism cases; in only 49 (4%) cases was congenital adrenal hyperplasia the most common (57.14%).

Conclusions

The results of this study provide useful clinical insights into the frequency of female hypogonadism in Basrah.

## Introduction

Female hypogonadism can be defined as deficient or abnormal functioning of the hypothalamic-pituitary-ovarian axis that clinically presents with disturbances in the menstrual cycle. Causes of female hypogonadism can be either congenital or acquired, and the defect can be in the hypothalamus, pituitary, or ovary. A precise history, physical examination, and select investigations can often help detect the location and type of the defect [1].

Puberty is a complex process affected by several environmental, metabolic, and genetic causes. Delayed puberty in females is a manifestation of hypogonadism in most patients [2]. Post-puberty, female hypogonadism typically manifests as estrogen deficiency, secondary amenorrhea, or hot flashes [1,3]. Hypogonadotropic hypogonadism is a clinical syndrome resulting from absent or inadequate pituitary gonadotropin secretion [3]. Hypogonadotropic hypogonadism is more common in males but can also occur in females. When associated with anosmia, it is known as Kallmann syndrome [1]. Female hypogonadism from pituitary disease presents with typical signs and symptoms, in addition to manifestations of associated pituitary hormonal insufficiencies [1]. Patients with primary conditions of the reproductive organs (congenital absence of the uterus, cervix, or vagina) can present with amenorrhea with or without signs of deficient estrogen [4]. Premature ovarian failure means failure before the age of 40 and often presents with oligomenorrhea or amenorrhea, elevated follicle-stimulating hormone (FSH), and low estradiol levels with hot flashes and vaginal dryness. Causes of premature ovarian failure could be genetic, autoimmune, post-surgical, or infection [5,6]. The terms “premature menopause” and “premature ovarian failure” used in the past for defining premature ovarian insufficiency are inaccurate because many patients receiving this diagnosis produced estrogen intermittently and ovulated, a few experienced intermittent menstrual cycles, and 5%-10% of cases conceived and had a normal pregnancy [7]. In Turner syndrome, XX and XY gonadal dysgenesis are common causes of hypergonadotropic hypogonadism in girls [8]. Hypothalamic amenorrhea or functional amenorrhea is a diagnosis of exclusion and can occur after any severe stressful conditions such as heavy exercise or intense emotional stress [9]. Differences in sex development (DSDs) represent a variety of congenital conditions of the urogenital tract and reproductive system affecting human sex determination and/or differentiation [10].

## Materials and methods

Study design and participants

This retrospective, single-center study was conducted at the Faiha Specialized Diabetes, Endocrine and Metabolism Center in Basrah, southern Iraq. To select eligible participants, we searched for diagnostic codes for hypogonadism between 2008 and May 2024 using keywords in the databases of the participating institutions. Female hypogonadism is an absence or deficient or abnormal function of the hypothalamic-pituitary-ovarian axis that clinically presents with disturbances in the menstrual cycle [1].

Hypogonadotropic hypogonadism features low or normal estradiol levels associated with low or normal levels of FSH, whereas hypergonadotropic hypogonadism is low or normal estradiol levels associated with high levels of FSH [8].

The inclusion criteria were women under 45 years old presenting with primary or secondary amenorrhea, delayed puberty, irregular menstrual cycle, infertility, and virilizing symptoms (hirsutism, hair loss, and deep voice).

Exclusion criteria were any patient diagnosed with polycystic ovary syndrome, diabetes mellitus, severe systemic illness such as thalassemia, chronic kidney disease, inadequate data or lack of evidence in medical records, and those diagnosed with pregnancy or menopause.

Data collection

We obtained data from the medical records of patients diagnosed with hypogonadism between January 2008 and May 2024. We searched using the following keywords: amenorrhea, hypogonadism, hypogonadotropic hypogonadism, hypergonadotropic hypogonadism, and DSD.

We recorded age, height, weight, body mass index (BMI), examination findings, and clinical diagnosis for all enrolled patients. We also recorded basal serum levels of gonadotropins, sex hormones, thyroid profiles, and prolactin, as well as outcomes of imaging studies when available. We recorded all data using a specifically designed program developed by the investigators based on the shared criteria.

Biochemical assessment

We used the fully automated chemiluminescence immunoassay kit Cobas e411 analyzer series (Roche Diagnostics, Germany) to measure hormones, including FSH, estradiol, prolactin, growth hormone (GH), thyroid-stimulating hormone (TSH), free thyroxine (FT4), total testosterone, adrenocorticotropic hormone (ACTH), and cortisol, with reference values presented in Table 1.

**Table 1:** Reference range of the measured hormones. TSH = thyroid-stimulating hormone; FT4 = free thyroxine; FSH = follicle-stimulating hormone; ACTH = adrenocorticotropic hormone

Hormones	Reference values
FSH	Males: 1–13 mIU/mL; Females: 2–12 mIU/mL
Estradiol	Females: 15–30 pg/mL
Prolactin	Males: 4–23 ng/mL; Females: 4–30 ng/mL
GH	0.01–0.97 ng/mL
TSH	0.27–4.2 μIU/mL
FT4	0.93–1.7 ng/dL
Total testosterone	Males: 265–1,200 ng/dL; Females: 10–55 ng/dL
ACTH	10–60 pg/mL
Cortisol	5–25 µg/dL

Statistical analysis

We analyzed the data using SPSS version 26 (IBM Corp., Armonk, NY, USA). The data were categorical and presented as frequencies and percentages.

## Results

**Figure 1:**
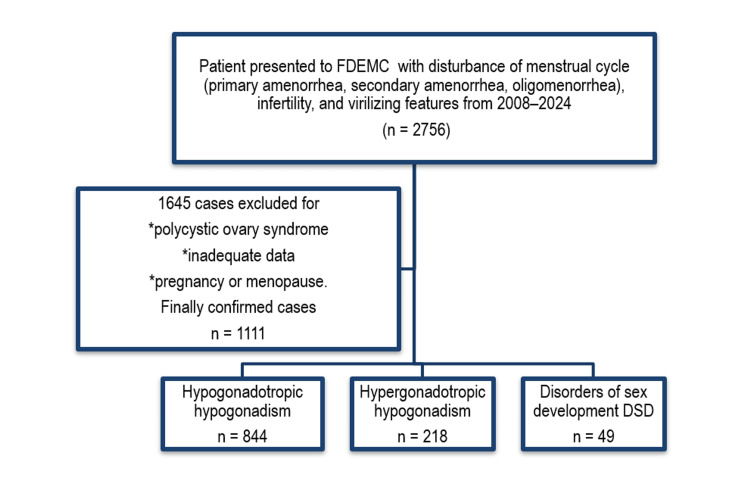
Flowchart of patients.

Among 2,756 initially enrolled cases, we categorized etiology in 1,111 cases while excluding 1,645 cases for diagnosis of polycystic ovary syndrome, pregnancy, menopause, or inadequate data. We divided the remaining 1,111 cases according to the type of hypogonadism into hypogonadotropic hypogonadism (844 cases), hypergonadotropic hypogonadism (214 cases), and DSDs (49 cases), as shown in Figure 1.

**Figure 2:**
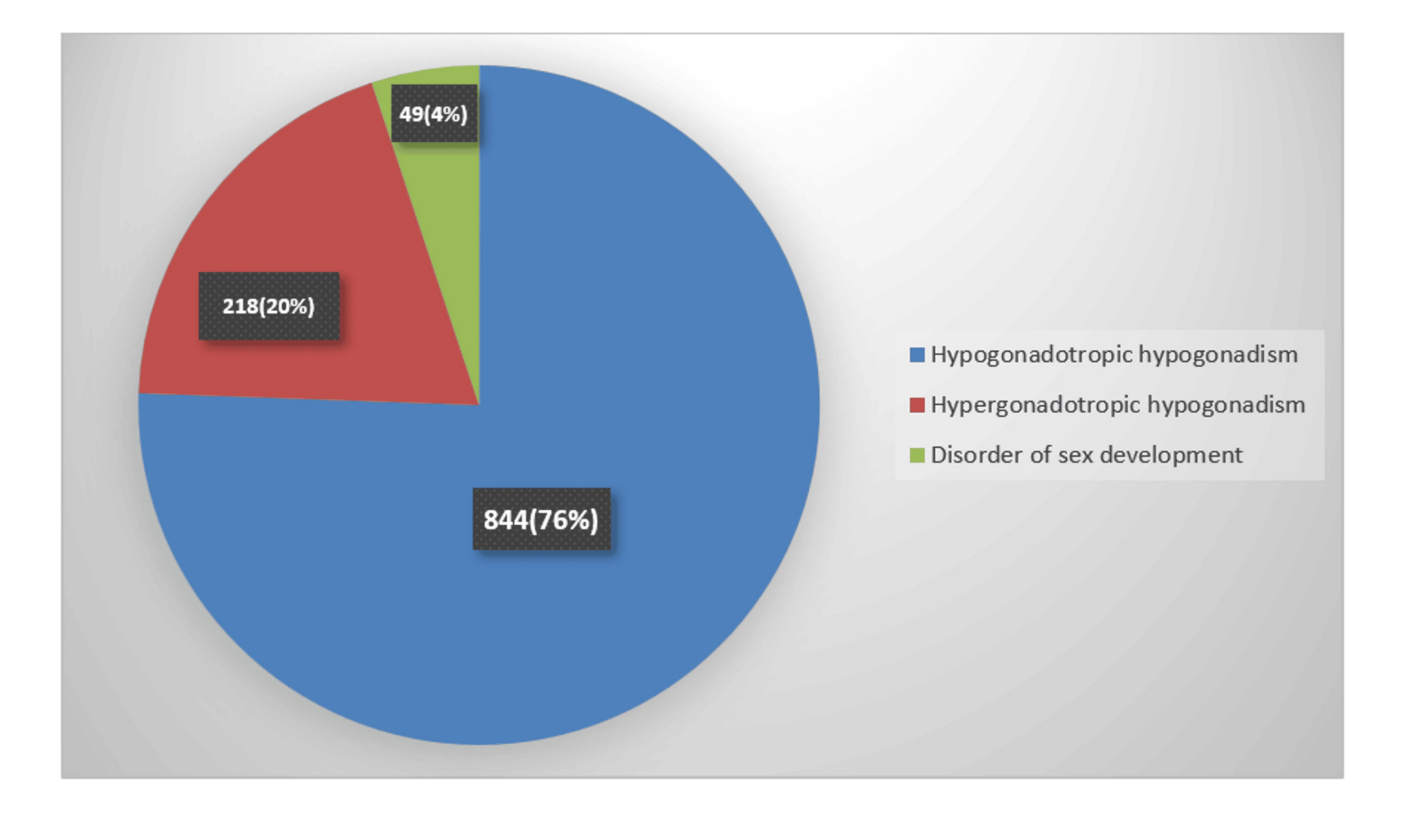
Distribution of female hypogonadism in Basrah among 1,111 patients.

Hypogonadotropic hypogonadism appeared in 844 (75.96%) cases, whereas 218 (19.6%) cases had hypergonadotropic hypogonadism. DSDs represented only 49 (4.4%) of the total number of cases, as shown in Figure 2.

Most patients in our study were of reproductive age, with a mean age ± SD of 30 ± 7 years. Most patients were overweight with a BMI of 27.5 ± 2.5 kg/m^2^, and 764 (69%) were married. Of the patients, 812 (73.08%) were unemployed, and 887 (79.83%) were of low economic status. Table 2 presents the demographic features of 1,111 female patients with hypogonadism.

**Table 2:** Demographic features of 1,111 female patients with hypogonadism. SD = standard deviation; BMI = body mass index

Variable	Mean ± SD or number (%)
Age (years)	30 ± 7
Height (m)	1.53 ± 0.05
Weight (kg)	65.3 ± 12.2
BMI (kg/m^2^)	27.5 ± 2.5
Marital status	
Married	764 (69)
Unmarried	347 (31)
Economic status	
Low	887 (79.83)
Medium	197 (17.73)
High	27 (2.4)
Occupation	
Unemployed	812 (73.08)
Employed	299 (26.91)

Regarding clinical presentations, in addition to the symptoms of the underlying etiology of hypogonadism, patients might present with one or more symptoms of estrogen deficiency. Of the patients, 999 (87.5%) presented with amenorrhea, 713 (71.37%) with secondary amenorrhea, 286 (25.7%) with primary amenorrhea, and 497 (44.73%) with oligomenorrhea. Infertility was present in 501 (44.85%) women with hypogonadism, 108 (9.66%) patients presented with delayed puberty, and virilizing symptoms such as hirsutism and hair loss were present in 28 patients. Furthermore, three girls showed hypogonadism when their families brought them for evaluation of unusually short stature, as shown in Table 3.

**Table 3:** Presentation of 1111 patients with hypogonadism. *: Patients might present with more than one symptom.

Presentation*	Number (%)
Primary amenorrhea	286 (25.7)
Secondary amenorrhea	713 (64.17)
Oligomenorrhea	497 (44.73)
Infertility	501 (44.85)
Delayed puberty	108 (9.66)

Of 844 females with hypogonadotropic hypogonadism, 402 (47.63%) were attributable to functional causes of hypogonadotropic hypogonadism, 203 (24%) had hypogonadism from pituitary and hypothalamic diseases, 69 (8.17%) from prolactinoma, 42 (4.97%) from acromegaly, 25 (2.96%) from empty sella syndrome, and 26 (3.08%) from hyperprolactinemia. Sixteen (1.89%) cases of hypogonadal women were the result of Sheehan syndrome, 13 (1.54%) from craniopharyngioma, nine (1.06%) from Cushing’s disease, and three (0.35%) from Kallmann syndrome. Adrenal disease also appeared in the etiology of female hypogonadotropic hypogonadism in 19 (2.25%) cases. Post-traumatic hypogonadism accounted for 12 (1.42%) cases, whereas in 208 (24.64%) cases, the causes remained unknown, as shown in Table 4.

**Table 4:** Etiology distribution of 844 patients with hypogonadotropic hypogonadism.

Diagnosis	Number (%)
Functional amenorrhea	402 (47.63)
Pituitary and hypothalamic	203 (24.05)
Prolactinoma	69 (8.17)
Hyperprolactinemia	26 (3.08)
Acromegaly	42 (4.97)
Empty sella syndrome	25 (2.96)
Sheehan syndrome	16 (1.89)
Craniopharyngioma	13 (1.54)
Cushing’s disease	9 (1.06)
Kallmann syndrome	3 (0.35)
Adrenal tumor	19 (2.25)
Post-traumatic	12 (1.42)
Unknown cause	208 (24.64)
Total	844 (100)

In 218 cases of hypergonadotropic hypogonadism, 170 (77.98%) had premature ovarian insufficiency, 47 (21.55%) had Turner syndrome, and only a single case (0.004%) had a virilizing ovarian tumor, as shown in Table 5.

**Table 5:** Distribution of the etiology in hypergonadotropic hypogonadism among 218 patients.

Diagnosis	Number (%)
Premature ovarian insufficiency and early Menopause	170 (77.98)
Turner syndrome	47 (21.55)
Ovarian tumor	1 (0.004)
Total	218 (100)

Overall, 49 patients had DSDs, distributed among 28 (57.14%) cases of congenital adrenal hyperplasia, 11 (22.4%) cases of gonadal dysgenesis, five (10.2%) cases of androgen insensitivity syndrome, and five (10.2%) cases of 5-alpha reductase deficiency, as shown in Table 6.

**Table 6:** Distribution of the etiology in females with disorders of sex development.

Diagnosis	Number (%)
Congenital adrenal hyperplasia	28 (57.14)
Gonadal dysgenesis	11 (22.4)
Androgen insensitivity syndrome	5 (10.2)
5-alpha reductase deficiency	5 (10.2)
Total	49 (100)

## Discussion

To our knowledge, this is the first study conducted in Iraq describing the causes of female hypogonadism. More than three-quarters of hypogonadal women had hypogonadotropic hypogonadism whereas one-fifth had hypergonadotropic hypogonadism. This large difference in proportion occurs for a variety of reasons and etiologies of female hypogonadotropic hypogonadism, starting from the hypothalamus to the ovaries and passing to the adrenal glands. On the one hand, every physical, psychological, or systemic illness may affect gonadotropin-releasing hormone (GnRH) secretion. On the other hand, the etiology of hypergonadotropic hypogonadism is limited to Turner syndrome and other rare causes of primary ovarian insufficiency [11].

Despite DSDs representing only a small percentage of the total number of cases, this is still a significant number of congenital defect cases that most likely end in hypogonadism and represent the most difficult cases to manage, as mentioned by Man et al. [10].

Most previous studies have analyzed the causes of hypogonadism separately, whether hypogonadotropic hypogonadism or hypergonadotropic hypogonadism, or have focused on a specific age group or specific diagnosis. Olivius et al. [12] studied the prevalence of female hypogonadism in hypopituitarism only. Kim et al. [13] studied etiology in primary amenorrhea only. Tang et al. [14] studied idiopathic hypogonadotropic hypogonadism only. Hence, our study may be the first of its type.

In the etiology of hypogonadotropic hypogonadism, we found that only a quarter of cases resulted from pituitary and hypothalamic pathology. This is inconsistent with Welt et al. who found that more than half of hypogonadism cases arose from hypothalamic and pituitary pathology [15].

Our study found that functional hypothalamic amenorrhea is a common cause of female hypogonadotropic hypogonadism, the results of which are similar to those of Shuflet et al. and Genazzani et al. [16,17]. Prolactinoma is predominant in pituitary causes of female hypogonadism, and this result is similar to that of Welt et al. [15].

Acromegaly, hyperprolactinemia, Sheehan syndrome, empty sella syndrome, and Kallmann syndrome are all rare causes of female hypogonadotropic hypogonadism, which in our study represented only a small percentage of cases [15,18,19]. Adrenal disorders were also documented to cause female hypogonadotropic hypogonadism in a small percentage of cases [20]. About a quarter of cases of female hypogonadotropic hypogonadism remained of unknown etiology (idiopathic). This result is compatible with the study by Silveira and Latronico, who found that about 40%-60% of hypogonadotropic hypogonadism cases were idiopathic [3], whereas Cham et al. showed that idiopathic hypogonadotropic hypogonadism constituted only 1-10 cases per 100,000 [21].

In women with hypergonadotropic hypogonadism, more than two-thirds of our patients had premature ovarian insufficiency, and approximately a quarter of patients had Turner syndrome. El-Dahtory et al. in Egypt found that 9.29% (80/860 cases) of infertile women had Turner syndrome, which was less frequent than in our study because they studied patients who presented with infertility only whereas our study included all presentations of female hypogonadism [22]. Ovarian tumor is a rare cause of hypergonadotropic hypogonadism. We found only a single case of such a diagnosis, which is consistent with the results of van Liempt et al. [23].

In our study, congenital adrenal hyperplasia was the most common cause of DSDs, representing more than half of cases, followed by gonadal dysgenesis in one-fifth of cases, and small percentages for androgen insensitivity syndrome and steroid 5-alpha reductase deficiency. These results are compatible with those of Man et al. [10].

Limitations

There are several limitations of this study. First, it was a single-center study. Second, it was a retrospective study, so history and examination findings could not be reviewed thoroughly. Third, there was some selection bias in the targeted patients because the study was conducted on referred patients to a tertiary center rather than on a general screening of the population. Hence, further large, comprehensive, multicenter studies are required to validate these findings.

## Conclusions

There are different etiologies of female hypogonadism in Basrah because it can be congenital or acquired and can result from defects in the pituitary, hypothalamus, or ovaries. Many cases of female hypogonadism remain undiagnosed, but the results of this study provide useful clinical insights into the frequency of the etiology of female hypogonadism in Basrah.
